# GraphPPL.jl: A Probabilistic Programming Language for Graphical Models

**DOI:** 10.3390/e26110890

**Published:** 2024-10-22

**Authors:** Wouter W. L. Nuijten, Dmitry Bagaev, Bert de Vries

**Affiliations:** 1 Department of Electrical Engineering, Eindhoven University of Technology, 5612 AE Eindhoven, The Netherlands; 2 GN Hearing, 5612 AB Eindhoven, The Netherlands

**Keywords:** Bayesian inference, factor graphs, nested models, probabilistic programming

## Abstract

This paper presents GraphPPL.jl, a novel probabilistic programming language designed for graphical models. GraphPPL.jl uniquely represents probabilistic models as factor graphs. A notable feature of GraphPPL.jl is its model nesting capability, which facilitates the creation of modular graphical models and significantly simplifies the development of large (hierarchical) graphical models. Furthermore, GraphPPL.jl offers a plugin system to incorporate inference-specific information into the graph, allowing integration with various well-known inference engines. To demonstrate this, GraphPPL.jl includes a flexible plugin to define a Constrained Bethe Free Energy minimization process, also known as variational inference. In particular, the Constrained Bethe Free Energy defined by GraphPPL.jl serves as a potential inference framework for numerous well-known inference backends, making it a versatile tool for diverse applications. This paper details the design and implementation of GraphPPL.jl, highlighting its power, expressiveness, and user-friendliness. It also emphasizes the clear separation between model definition and inference while providing developers with extensibility and customization options. This establishes GraphPPL.jl as a high-level user interface language that allows users to create complex graphical models without being burdened with the complexity of inference while allowing backend developers to easily adopt GraphPPL.jl as their frontend language.

## 1. Introduction

Probabilistic programming languages (PPLs) are designed to simplify the complexities of probabilistic inference. They offer a high-level modeling language and an inference engine that automates various inference algorithms, including Bayesian methods. While PPLs often facilitate Bayesian inference, they can also support a broader range of probabilistic models and inference algorithms. Effective use of these PPLs usually requires extensive knowledge of the underlying principles, but recent advances in PPLs, such as NumPyro [1] and Turing.jl [2], aim to reduce this burden on the user. These languages have significantly advanced the field of probabilistic reasoning, enabling applications across diverse domains such as toxicology [3], geophysics [4], and cognitive sciences [5]. They provide users with powerful inference results without the need to customize inference software, allowing researchers without deep knowledge of probabilistic information processing to effortlessly analyze observed data. With the help of PPLs, users only need to interact with a high-level interface and define the generative models that govern data generation.

Once the model has been defined, the extra difficulty lies in achieving feasible inference within limited computational resources. Numerous methods to automate the inference process have been suggested, such as sampling-based methods [2,6,7] or variational optimization-based methods [8,9,10]. However, most automated inference solutions are intertwined with their model specification component. This makes it challenging to use the same probabilistic model with different inference methods and necessitates redefining the model for each specific inference method.

This paper addresses this issue by introducing GraphPPL.jl, a PPL that enables users to define a probabilistic model without making assumptions about a specific inference process. The frontend can generate a model as a graph and provides a plugin system to add inference-specific information if needed. To illustrate this capability, GraphPPL.jl includes a variational inference plugin, which injects additional information into the standard model structure and enables support for variational inference methods, such as the Constrained Bethe Free Energy (CBFE) optimization process in RxInfer [10].

The selection of CBFE minimization to demonstrate the capabilities of GraphPPL.jl is deliberate. It has been demonstrated that numerous traditional inference approximation methods can be expressed as the minimization of a CBFE functional. Nonetheless, GraphPPL.jl does not impose any particular assumptions regarding the inference process and can potentially act as a frontend for various other inference techniques, including HMC [11], NUTS [12], and more. GraphPPL.jl outputs a data structure representing the generative model without relying on any inference backend. As far as we know, no other PPL currently provides this feature.

Furthermore, GraphPPL.jl facilitates the definition of nested models, allowing large and complex models to be built from smaller, simpler ones. This is crucial because the exponential increase in computing power has made Bayesian inference, once extremely costly, feasible on standard computer chips. This advancement has broadened the range of generative models, with recent techniques scaling to thousands of variables [2,13], making the specification of such large models even more challenging. When dealing with complex systems, dividing them into separate independent modules (e.g., functions or classes in programming languages) is common practice. However, a concept similar to modularity in probabilistic programming is still underdeveloped, and GraphPPL.jl aims to bridge this gap.

The structure of this paper is outlined as follows:

- Section 2 reviews factor graphs, variational Bayesian inference, and CBFE minimization. Additionally, we discuss why CBFE minimization on a factor graph is considered a general, customizable inference method.
- Section 3 discusses related works.
- Section 4.2 delves into the core design philosophy of GraphPPL.jl and explains the rationale for implementing the CBFE minimization plugin as the default in GraphPPL.jl.
- Section 4.3 introduces the @model macro and syntax, which serves as the primary entry point for creating any GraphPPL.jl model.
- Section 4.4 showcases how models defined with the @model macro can be reused in larger models, thereby adding modularity to the language.
- Section 4.7 presents the @constraints macro, which specifies factorization and functional-form constraints on the variational posterior for the inference engine. We define a clear and intuitive constraint language. Both @model and @constraints exemplify the integration of models specified within GraphPPL.jl with a particular inference backend.
- Section 5 demonstrates the integration of GraphPPL.jl in the RxInfer.jl inference ecosystem with an inference example.

## 2. Background

This section aims to provide an overview of Bayesian inference, variational inference, factor graphs, and CBFE minimization. We regard understanding these concepts as essential to appreciating the language design of GraphPPL.jl. However, this is not meant to review the topics fully. Instead, we refer interested readers to [14] where these concepts are discussed in more detail.

### 2.1. Bayesian Inference

Consider a factorized generative model p(x,z)=p(x|z)p(z) with observations x and unobserved latent states z. Note that the factorization of the model p(x,z) can be reversed by Bayes’ rule as

(1) $$p\left(x,z\right)=\underset{\mathrm{likelihood}}{\underset{︸}{p(x\;|\;z)}}\underset{\mathrm{prior}}{\underset{︸}{p(z)}}=\underset{\mathrm{posterior}}{\underset{︸}{p(z\;|\;x)}}\underset{\mathrm{evidence}}{\underset{︸}{p(x)}}\;,$$

where the evidence can be computed by

(2) p(x)=∫p(x,z)z.

In a probabilistic modeling context, the first factorization postulates the model as a product of likelihood and prior. Bayesian inference aims to derive the second factorization as the product of posterior and evidence. Both the Bayesian posterior and the model evidence are very interesting quantities, as the posterior describes the distribution over latent states z after observing data x, and the model evidence quantifies the model’s performance in explaining the observed data. Unfortunately, the integration over latent states in Equation (1) quickly becomes intractable, as the computational complexity of the integration is exponential in the dimensionality of z. Therefore, much effort has been devoted to finding a manageable and accurate approximation of Bayesian inference when computing the model evidence, which is computationally not feasible with available resources. One of the solutions involves further factorizing the likelihood and prior distributions, a concept that we will explore next.

### 2.2. Factor Graphs

In this section, we explore factor graphs and their application in expressing generative models. For simplicity, we write our generative model as p(s) without explicitly decomposing the variables s into z and x. Assume that we can further factorize our model as follows:

(3) $$p\left(s\right)=\prod_{i}^{m}f_{i}\left(s_{i}\right),$$

meaning *p* adheres to a factorization into *m* factor functions $\left\{f_{a},f_{b},\cdots f_{N}\right\}$, where each of the factor functions is a function over a subset of the variables s. For example, we could factorize a distribution over four variables as follows:

(4) $$p\left(s_{1},s_{2},s_{3},s_{4}\right)=f_{a}\left(s_{1}\right)f_{b}\left(s_{1},s_{2},s_{3}\right)f_{c}\left(s_{2},s_{4}\right)f_{d}\left(s_{3}\right)f_{e}\left(s_{4}\right).$$

**Figure 1:**
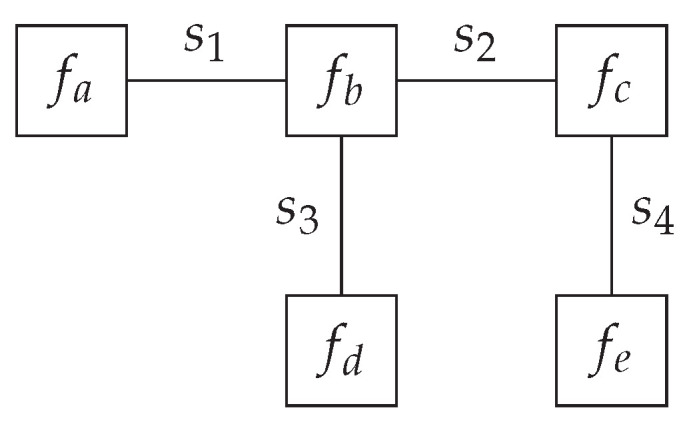
Forney-style factor graph corresponding to the factorization in Equation (4).

If the generative model adheres to a similar factorization, we can draw a computation graph of the generative model, where the vertices represent the factor functions and the edges represent the variables. This visual representation of a factorized generative model is called a Forney-style factor graph (FFG) [15]. An FFG is mathematically represented as a graph G=(V,E), where V is a set of vertices, and E is a set of edges. We use (a,b,c…) as indices for vertices and (i,j,k…) as indices for edges. An example of the FFG corresponding to Equation (4) can be seen in Figure 1.

Computing the Bayesian posterior and marginal distributions in a factorized distribution is computationally easier than the integration problem in Equation (1). This is because we can use the factorization in Equation (4) to reduce the dimensionality of the integration as follows:

(5) $$\begin{array}{cc} p(s_{2})= & \int p\left(s_{1},s_{2},s_{3},s_{4}\right)ds_{1}ds_{3}ds_{4}\\ = & \int f_{a}\left(s_{1}\right)f_{b}\left(s_{1},s_{2},s_{3}\right)f_{c}\left(s_{2},s_{4}\right)f_{d}\left(s_{3}\right)f_{e}\left(s_{4}\right)ds_{1}ds_{3}ds_{4}\\ = & \underset{\vec{\mu }\left(s_{2}\right)}{\underset{︸}{\int f_{a}\left(s_{1}\right)\int f_{b}\left(s_{1},s_{3},s_{3}\right)f_{d}\left(s_{3}\right)ds_{3}ds_{1}}}\cdot \underset{\overset{\leftarrow }{\mu }\left(s_{2}\right)}{\underset{︸}{\int f_{c}\left(s_{2},s_{4}\right)f_{e}\left(s_{4}\right)ds_{4}}}. \end{array}$$

**Figure 2:**
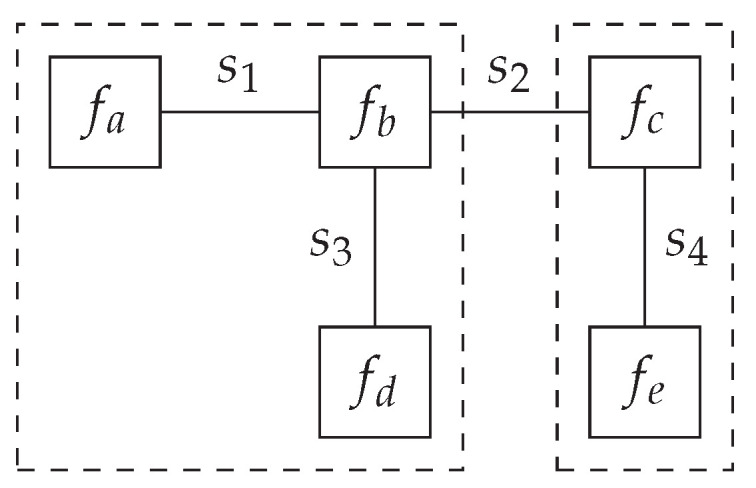
FFG representation of the computation of the marginal distribution over $s_{2}$ by computing and multiplying the marginal distributions of two submodels.

Here, we can see that the integral to compute $p(s_{2})$ can be split into two separate integration operations. We can also visually represent this separation in an FFG, as illustrated in Figure 2. Here, we see that $\vec{\mu }\left(s_{2}\right)$ corresponds to computing the marginal distribution of $s_{2}$ in the subgraph enclosed by the left dotted box. Similarly, $\overset{\leftarrow }{\mu }\left(s_{2}\right)$ corresponds to marginalizing over $s_{4}$ in the subgraph enclosed by the right dotted box. This simple example illustrates that we can group individual factor nodes together. After marginalization over the internal variables in this group, we obtain the marginal distributions over the variables that connect to nodes outside of this group. We call these “boundary-crossing” edges the Markov blanket of a group of factors. Visually, we can represent this by drawing a box around a group of factor nodes, as shown in Figure 2. This process is called “closing the box” [15] and allows us to represent any FFG as a hierarchy of nested models. By iteratively defining FFGs and their corresponding Markov blankets, we can use previously defined FFGs as nodes in new factor graphs, giving us a modular composition of the final FFG.

### 2.3. Variational Inference

In Bayesian inference, we are interested in determining posterior distributions over unobserved latent states given observations, adhering to some generative model. Variational inference is concerned with approximating p(z|x) by introducing a candidate distribution q(z) and minimizing the variational free energy (VFE):

(6) $$\begin{array}{cc} F\left[q\right] & ≜\int q\left(z\right)\log\frac{q(z)}{p(x,z)}dz\\ & =\underset{D_{KL}\left[q(z)\;|\;\;|\;p(z\;|\;x)\right]}{\underset{︸}{\int q\left(z\right)\log\frac{q(z)}{p(z|x)}dz}}-\underset{\log\;\mathrm{evidence}}{\underset{︸}{\log p(x)}}. \end{array}$$

Here, $D_{KL}$ is the Kullback–Leibler divergence [16], which is a measure of how one probability distribution *q* is different from a reference probability distribution *p*. In other words, it measures how much information is lost when *q* is used to approximate *p*, making it a suitable measure for approximate inference. By choosing a variational family of tractable distributions Q, we can define the variational posterior distribution as follows:

(7) $$q^{*}=\arg\min_{q\in Q}F\left[q\right]$$

From the VFE in Equation (6), we can see that $q^{*}$ will approximate p(z|x), and $F\left[q^{*}\right]$ will approximate logp(x). In this way, variational inference transforms the integration problem in Equation (1) into an optimization problem in Equation (7). We can then apply traditional (un)constrained optimization methods to Q to find an approximate posterior distribution. We refer readers to [17] for an extensive review of variational inference.

### 2.4. Constrained Bethe Free Energy

The factorization of Equation (3) also has consequences for the VFE since the denominator in Equation (6) now becomes a factorized distribution. It is common practice to factorize the candidate distribution q(s) similarly to the generative model. This means that when we draw the FFG representation of the generative model, we assign candidate functions, which we call beliefs, to the nodes and edges of the graph, which, when multiplied, correspond to our candidate distribution. Therefore, we define the family $Q_{G}$ as follows:

(8) $$q\left(s\right)\in Q_{G}\Rightarrow q\left(s\right)=\prod_{a\in V(G)}q_{a}\left(s_{a}\right){\left(\prod_{i\in E(G)}q_{i}\left(s_{i}\right)\right)}^{-1}.$$

Here, $q_{a}\left(s_{a}\right)$ are the node-wise marginal beliefs defined for every node *a*, and $q_{i}\left(s_{i}\right)$ are the edge-wise marginal beliefs defined for every edge *i*. The division of all edge beliefs is an artifact of the fact that we always count every edge twice in the first multiplication; hence, we need a correcting term [18]. Suppose both the generative model and variational distribution follow these factorizations. In that case, it can be shown that the VFE can be rewritten as the Bethe Free Energy (BFE) [19], which is defined as

(9) $$\begin{array}{cc} F\left[q\right]≜ & \sum_{a\in V(G)}\underset{F[q_{a}]}{\underset{︸}{\int q_{a}\left(s_{a}\right)\log\frac{q_{a}\left(s_{a}\right)}{f_{a}\left(s_{a}\right)}ds_{a}}}+\sum_{i\in E(G)}\underset{H[q_{i}]}{\underset{︸}{\int q_{i}\left(s_{i}\right)\log\frac{1}{q_{i}\left(s_{i}\right)}ds_{i}}}. \end{array}$$

The BFE calculates a local VFE term for each node in the FFG, allowing us to minimize the VFE at each node individually. However, because the BFE divides the global optimization problem into multiple local optimization problems, it is necessary to impose constraints on the variational posterior q(s) to ensure that, after optimizing the BFE, the result is still a valid probability distribution. The required constraints are normalization and marginalization constraints:

(10) $$\begin{array}{cc} \; & \int q_{a}\left(s_{a}\right)ds_{a}=1,\mathrm{for}\;\mathrm{all}\;a\in V\left(G\right)\\ \; & \int q_{a}\left(s_{a}\right)ds_{a\setminus i}=q_{i}\left(s_{i}\right),\mathrm{for}\;\mathrm{all}\;a\in V\left(G\right),i\in E\left(G\right). \end{array}$$

Minimization of the BFE over a constrained variational family is called Constrained Bethe Free Energy (CBFE) minimization, and it can be shown that the stationary points of the BFE correspond to the local minima of the belief propagation algorithm [20,21]. By introducing additional constraints on the variational posterior, many popular inference algorithms are recognized as instances of CBFE minimization [14]. For example, we obtain the mean-field algorithm by further constraining the node beliefs $q_{a}\left(s_{a}\right)$ to be fully factorized. Therefore, a PPL that allows users to specify a CBFE of their generative model gives the user a powerful language to define generative models and influence the inference results.

## 3. Related Works

Probabilistic programming languages aim to provide users with an intuitive way to create probabilistic models for inference. In general, existing PPLs are designed with the models on which we can perform efficient inference in mind. Although this places constraints on the expressiveness of the modeling language, this approach has resulted in successful languages such as BUGS [6,22], STAN [7,23], and CHURCH [24]. In some languages, such as IBAL [25], inference details are part of the model specification, further intertwining model definition and inference implementation. Over time, numerous probabilistic programming libraries have emerged, each contributing unique perspectives to the field [24,25,26,27,28]. In recent years, the increasing popularity of *Python* and its rich deep learning community have paved the way for PPLs in *Python*, using a deep learning engine as a backend, such as Pyro [27] and TensorFlow Probability [29]. For a more comprehensive review of probabilistic programming languages, we refer readers to [30].

An effort was made to construct a universal PPL that does not depend on inference details through Turing.jl [2], a PPL in the *Julia* [31] language aimed at providing universal inference through sampling. With the implementation of DynamicPPL.jl language, considerable effort was made to ensure the separation between the modeling language and the inference process. The increasing popularity of Turing.jl suggests the need for a universal modeling language that is not restricted by the details of inference. Turing.jl also makes an effort toward modularity; with the @submodel macro, existing Turing.jl models can be called within larger models. However, we can only fit the submodel in the forward generative direction, so this is not a general solution for modular probabilistic programming.

The utility of functions in traditional programming languages is well recognized, primarily due to their ability to facilitate code reuse, reduce errors in program definitions, and minimize the number of code lines. This concept parallels the realm of nested probabilistic models within PPLs. The necessity for nested PPLs arises from their potential to conserve lines of code, decrease errors in model definitions, and enhance reusability, mirroring the benefits offered by functions in conventional programming languages. This notion of modularity and reusability in programming is not just a matter of convenience or good software engineering practice; it also resonates with our understanding of intelligent systems, both biological and artificial.

## 4. The GraphPPL.jl Engine

In this section, we elaborate on the philosophy and details of the GraphPPL.jl (https://github.com/ReactiveBayes/GraphPPL.jl (accessed on 18 September 2024), documentation available at https://reactivebayes.github.io/GraphPPL.jl/stable/ (accessed on 18 September 2024)) package, explaining the design choices and highlighting its distinguishing features. We also explain the rationale behind focusing specifically on the CBFE for the inference backend integration.

We implemented GraphPPL.jl in the *Julia* [31] programming language. The selection of *Julia* as the programming language is primarily due to two key factors. First, *Julia* offers high performance tailored for scientific computing. Given the computational demands of Bayesian inference, a performance-centric language is essential. While the PPL itself may not be heavily impacted by this computational load, having both the user language and the inference backend in the same language provides considerable benefits. Second, *Julia*’s meta-programming capabilities enable us to create a custom syntax that is converted into standard *Julia* code. This allows us to enhance the *Julia* language with the specific features and operators required for probabilistic programming.

### 4.1. Representing Graphical Models

GraphPPL.jl offers a frontend for defining probabilistic models, providing the flexibility to accommodate various inference backends. These probabilistic models are represented as graphs, enabling visualization and inspection of their properties without committing to a particular inference method. Models can be stored in memory or transmitted over the internet to a standalone device for later use. The graph-based representation allows models to be broken down into smaller components, or submodels, which can be reused in more complex hierarchical probabilistic models. The choice of a factor graph representation allows for an analysis of the created generative model, and inference backends can match subgraphs against known models to employ faster and more tractable inference procedures [14].

Nonetheless, GraphPPL.jl recognizes that the main objective is to perform inference on the specified model. To facilitate the integration of inference methods, GraphPPL.jl employs a plugin system that allows for the addition of extra information to the graph required by specific inference backends. For instance, this information might include specific hyperparameters for sampling-based approaches like HMC or constraints for variational inference methods. As an example of such seamless integration, GraphPPL.jl integrates with a particular inference method called the Constrained Bethe Free Energy (CBFE) framework. In Section 4.7, we will showcase the integration of variational constraints in GraphPPL.jl, allowing CBFE definition.

### 4.2. Language Philosophy

In designing GraphPPL.jl, we adopted a usability-centric approach that focuses primarily on providing high-level model specification capabilities that integrate with various inference operations without depending on a specific inference backend. With this usability-centric approach, we aim to balance expressiveness, ease of use, and learnability, ensuring that novice and experienced users can leverage the language effectively without being burdened by a steep learning curve during their initial acquaintance.

The philosophy of GraphPPL.jl rests on a few requirements:

- Creating probabilistic models with GraphPPL.jl should resemble drafting a mathematical description of a generative model. Similar to Turing.jl [2] and BUGS [22], the core language of a GraphPPL.jl model should closely match the mathematical representation of the generative model.
- GraphPPL.jl models should be as readable as *Julia* programs. Drawing inspiration from PyTorch [32] and their approach of treating ’deep learning models as *Python* programs’, we want GraphPPL.jl models to have the same readability and feel as *Julia* programs. As a result, any GraphPPL.jl model should be usable as a component within a larger GraphPPL.jl model.
- A materialized GraphPPL.jl model should be compatible with various inference backends; for example, CBFE minimization. The model should be extendable to inject all required information for any inference backend to perform Bayesian inference.

### 4.3. The Model Specification Language

To specify a model in a language that is as close as possible to the mathematical representation of a model, we employ *Julia*’s [31] powerful meta-programming functionality to create our own syntax. By creating our own syntax using the @model macro, we encapsulate all the necessary logic for constructing a GraphPPL.jl model. A consequence of using *Julia*’s macro functionality is that we extend the syntax of *Julia*, meaning our language accepts any regular *Julia* code while extending the *Julia* syntax to allow probabilistic modeling. We begin by constructing a model that characterizes a sequence of coin tosses to introduce the model syntax. The model for *n* coin tosses is defined in Equation (11).

(11) $$\begin{array}{cc} \theta & ∼\mathrm{Beta}(1,1)\\ y_{i} & ∼\mathrm{Bernoulli}(\theta ),\;\mathrm{for}\;\mathrm{all}\;i\in [1.\;.n] \end{array}$$

The @model syntax accepts a *Julia* function. The function’s arguments are all data structures external to the model, such as the data, but we could also include priors or hyperparameters. These arguments are called interfaces because they connect the model’s exterior with its interior. Since our model receives data *y*, we begin our model definition by creating a function: @model **function** coin_toss(y).

**Figure 3:**
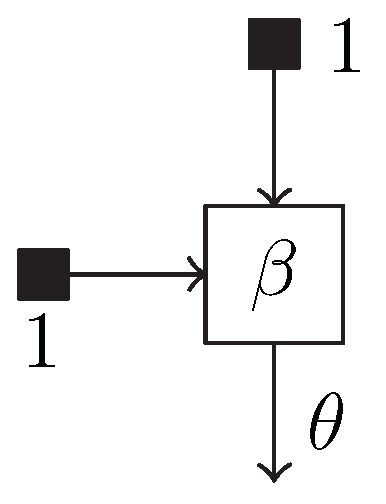
Subgraph created by the statement *θ* ~ Beta(1, 1) in the @model macro.

The @model macro exposes a GraphPPL.jl-specific operator: the ~. This operator creates a new factor node and a variable in the factor graph. For example, the statement *θ* ~ Beta(1, 1) creates a Beta factor node and a *θ* variable, along with two variables representing the constant 1. It connects these to the Beta node. The subgraph created by this statement can be seen in Figure 3.

The ~ operator in GraphPPL.jl checks whether the factor being created is stochastic or deterministic. If it is deterministic and the arguments are known at model construction time, GraphPPL.jl will not create a factor node but will execute the function and return the result. For example, calling a ~ norm([1, 2, 3]) in GraphPPL.jl will transform to a = norm([1, 2, 3]), making the constant a available for the rest of the model specification.

As the model macro accepts *Julia* syntax, we can combine the ~ operator with regular *Julia* syntax to write the rest of the model. The complete model for the coin tosses defined in Equation (11) can be found in Code Block 1.


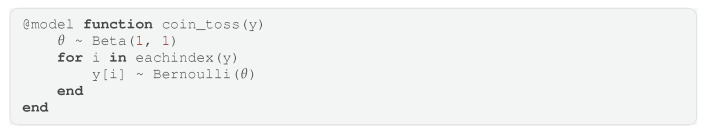

**Code Block 1.** GraphPPL.jl code for the coin toss model defined in Equation (11).

The ~ operator is not the only way to create factor nodes and variables in the underlying factor graph; we can use the := operator, which is an alias of the ~ operator, to denote deterministic relations. Furthermore, GraphPPL.jl supports compound statements and will unroll any statement on the right-hand side of the ~ operator to create all factor nodes. To illustrate this, we implement the Gaussian-with-Controlled-Variance (GCV) [33] model, a fundamental building block of the widely used hierarchical Gaussian filter [34]. The GCV model, with inputs x, *ω*, *κ*, and z, is defined as

(12) y∼N(x,exp(κ∗z+ω)).

**Figure 4:**
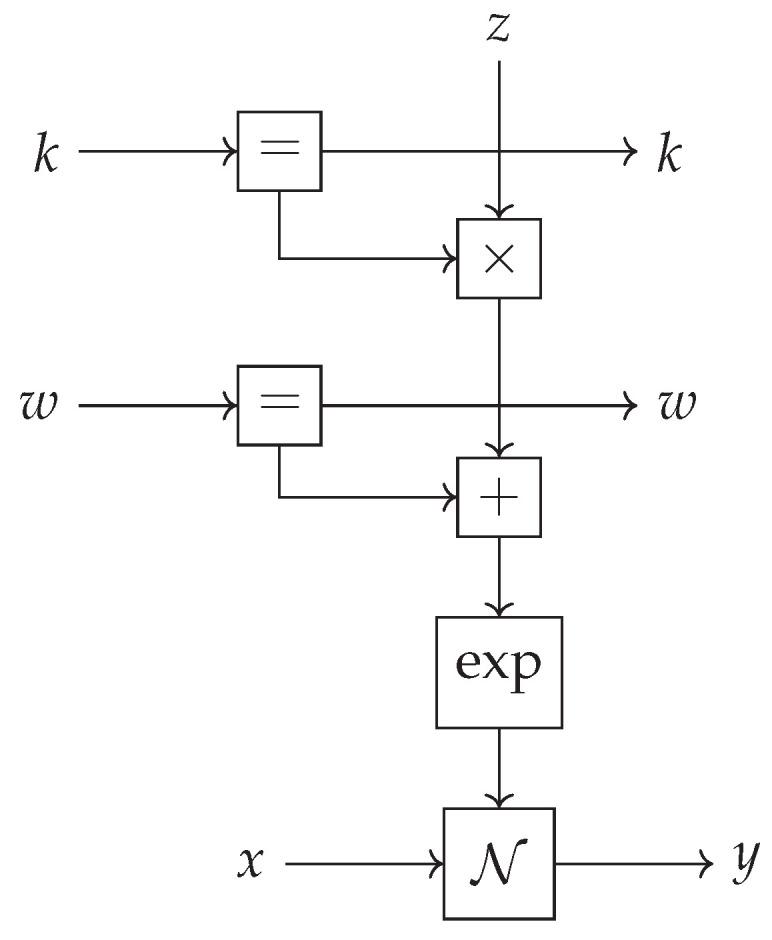
A Forney-style factor graph representation of the Gaussian-with-Controlled-Variance (GCV) model created by the model definitions in Code Blocks 2 and 3.

Two GraphPPL.jl model definitions, both defining the same model, can be seen in Code Blocks 2 and 3. The model definition in Code Block 2 uses a compound statement to create Normal, exp, +, and ∗ factor nodes in the same line. The model definition in Code Block 3 uses the := as an alias for the ~ operator to denote deterministic relations. Both model definitions create the same factor graph, as shown in Figure 4. For both models, x, *ω*, *κ*, and z are inputs to the model, and y is the output. We use these five variables as interfaces, which are the arguments of the model function.


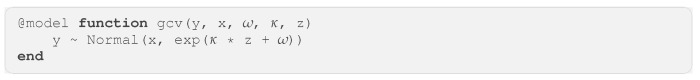

**Code Block 2.** The GCV model from Equation (12) implemented with compound statements.

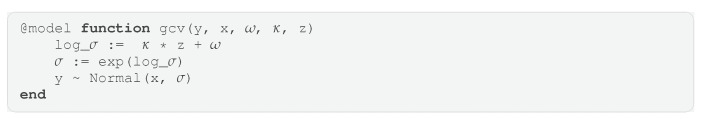

**Code Block 3.** The GCV model from Equation (12) implemented with deterministic statements.

### 4.4. Modular Definition and Usage of Models

**Figure 5:**
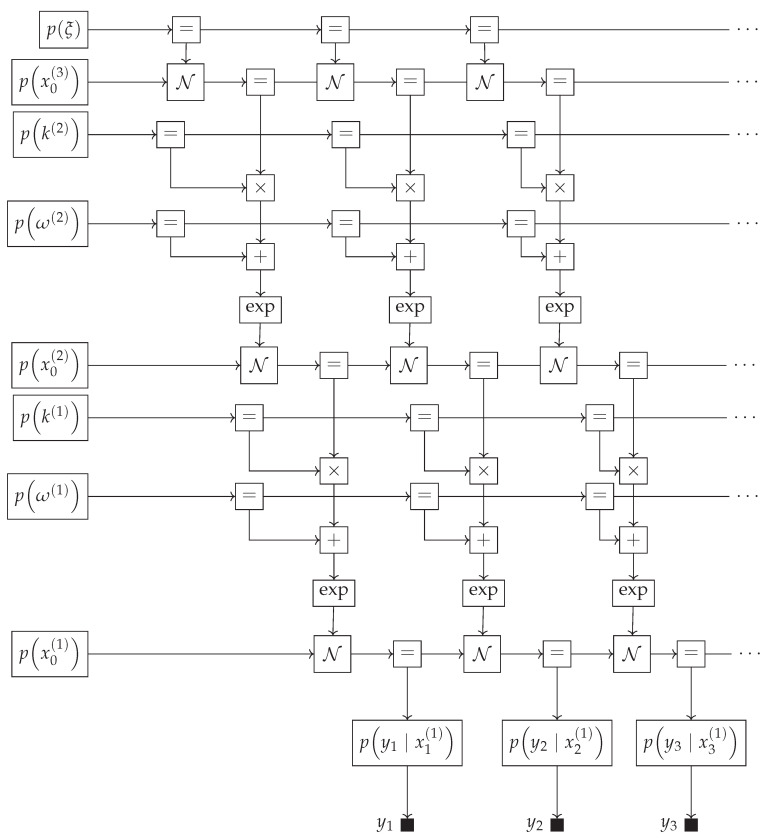
A 3-layer hierarchical Gaussian filter drawn as a Forney-style factor graph. The first 3 time steps are depicted.

**Figure 6:**
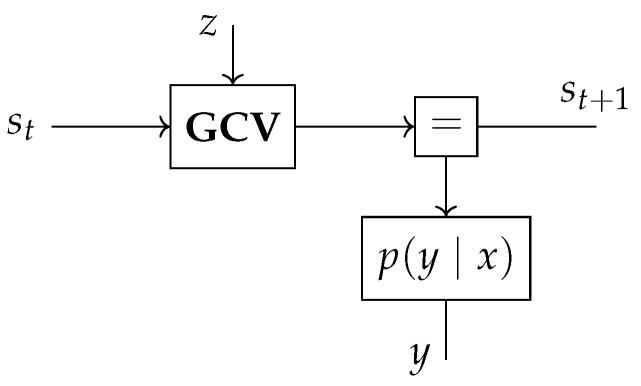
The Gaussian-with-Controlled-Variance Likelihood Model (GCV-LM) submodel.

Generative models for real-world processes can become unwieldy and large. An example can be seen in Figure 5, where we visualize a hierarchical Gaussian filter of depth 3, and the factor graph representation of the generative model for the first three data points is depicted. While we only visualize three data points, we usually want to incorporate hundreds to thousands of data points [14,35]. The three-level HGF is most often used in the literature [34,35]; however, the model is not limited to three layers. Recreating this model in previously discussed PPLs involves manually creating all nodes, a cumbersome and error-prone process that results in unnecessarily long model specifications. In contrast, in GraphPPL.jl, we can define models and use them as submodels of other GraphPPL.jl models. An example of this can be seen in Code Block 4, which uses the gcv submodel to chain a Gaussian-with-Controlled-Variance state transition with a Gaussian likelihood model. Note that when invoking gcv, we supply all but one interface through named keyword arguments. GraphPPL.jl can recognize which interface to the gcv submodel is missing and will attach this interface to the x_next variable that is on the left-hand side of the ~ operator. The factor graph representation of this model can be seen in Figure 6. For visual clarity, we have grouped *k*, *w*, and $x_{t}$ as the state variable $s_{t}$. Although this model describes a complex state transition and a likelihood model, the FFG is remarkably easy to read.


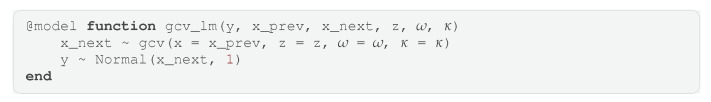

**Code Block 4.** Model chaining a gcv submodel with a Gaussian likelihood.

With the gcv and gcv_lm models defined, we can now simplify the HGF model shown in Figure 5 to use these submodels. The GraphPPL.jl code for creating the HGF model can be seen in Code Block 5. For each data point in *y*, we compute state transitions using the gcv and gcv_lm submodels to fully specify the likelihood model. Note that the number of lines of GraphPPL.jl code we have written so far is still fewer than 20. In this definition of hgf, we can pass constants or distributions as arguments *ξ*, *ω*, and *κ*, which will serve as either parameters or priors to these parameters. Note that in Code Block 5, we have used the new syntax, which creates a new variable in a vector of random variables if it does not exist. With this syntax, we can specify an observation through a state transition and create both variables on the same line.

**Figure 7:**
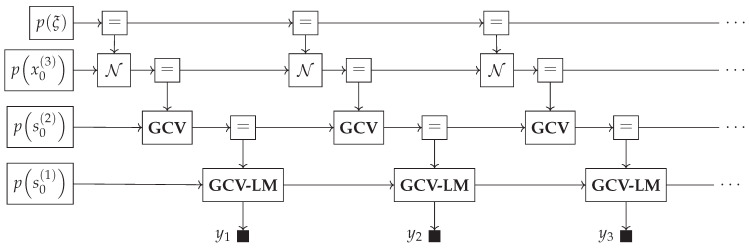
The hierarchical Gaussian filter shown in Figure 5 drawn as a composite model using the **GCV** and **GCV-LM** submodels.

The FFG of the HGF model using our defined submodels can be viewed in Figure 7. In this graph, we obtain a clearer picture of the computation flow and can better communicate to readers the intentions of the model and the modeling decisions made. More importantly, if we wanted to use a different likelihood model instead of the Gaussian likelihood specified in Code Block 4, we would only have to change this submodel, and every instance of gcv_lm in the hgf model would change accordingly. This contrasts with widely used PPLs, where the likelihood model for every timestep must be changed individually.

The nested model specification in GraphPPL.jl enables researchers to easily build intricate models, facilitating the exploration and creation of sophisticated probabilistic models without sacrificing readability or maintainability. This hierarchical model definition and composition stand out as defining features, representing a significant leap in usability and model organization, aligning with established programming design principles such as the Single Responsibility Principle [36].


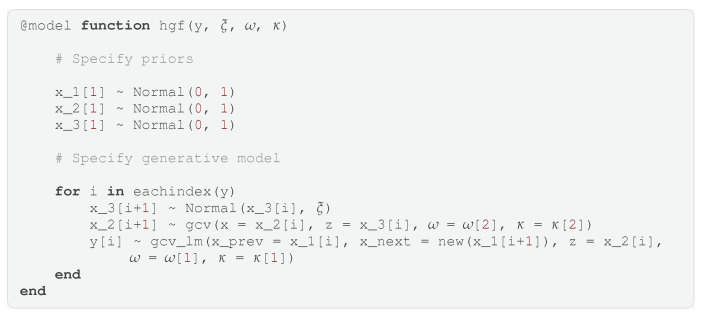

**Code Block 5.** The hierarchical Gaussian filter defined in GraphPPL.jl using the gcv and gcv_lm submodels.

### 4.5. Example: Bayesian Neural Network

Now that we have introduced the core functionality of the language, we can demonstrate an advanced example. Although GraphPPL.jl is not a language specifically designed for neural networks, it is expressive enough to define Bayesian neural networks in a few lines of code. As in the previous section, we build a larger model starting with smaller submodels. First, we start by defining a neural_dot submodel that takes a vector input and a vector of weights w, returning the dot product between input and w while applying an activation function, as shown in Code Block 6. This, together with the definition of a weight vector, defines an artificial neuron, as seen in Code Block 7.


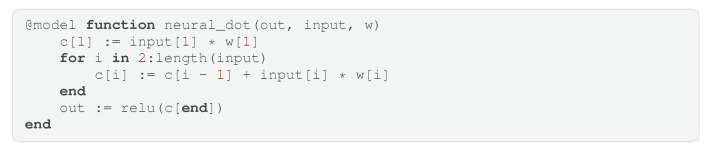

**Code Block 6.** Dot product with nonlinearity applied as the basic building block of a neural network.

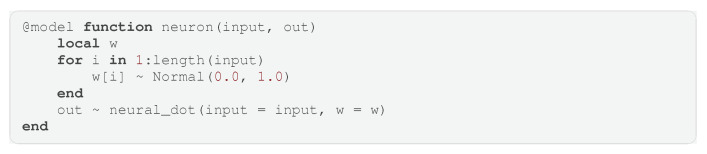

**Code Block 7.** Artificial neuron.

We can now define a single fully connected layer consisting of neurons that share the same input and produce a single output element (Code Block 8). With this defined, we can create a multilayer perceptron or fully connected neural network. The code to create a four-layer Bayesian neural network can be seen in Code Block 9. This example again highlights how GraphPPL.jl can construct intricate probabilistic models by combining smaller, simpler ones.


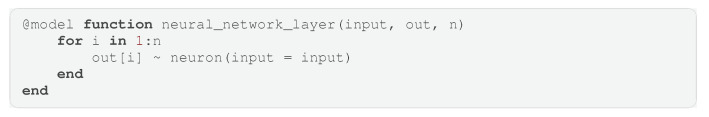

**Code Block 8.** Fully connected neural network layer defined as a composition of neurons.

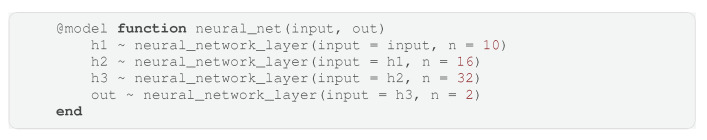

**Code Block 9.** Example of a multilayer perceptron.

### 4.6. Extensibility

GraphPPL.jl does not operate only with a predetermined set of factor nodes. Instead, it contains an extensible architecture that relies on *Julia*’s multiple dispatch functionality to determine when to materialize a factor node for a function and when to evaluate a function with the supplied arguments deterministically. By specifying whether a function is deterministic or stochastic, developers of inference backends can introduce new nodes to be integrated into probabilistic modeling.

The power of this design becomes evident when considering the Normal distribution used in Code Block 2. Rather than being part of the native GraphPPL.jl, the Normal distribution is part of an extension that becomes available upon loading the Distributions.jl package. This extension defines the essential functionality required to create a node for each distribution in Distributions.jl, expanding the range of distributions accessible for probabilistic modeling without explicitly relying on Distributions.jl out of the box.

Some inference backends (such as ReactiveMP.jl [37]) might expose different implementations of well-known distributions for computational efficiency. For example, ReactiveMP.jl exposes four implementations of the Normal distribution: NormalMeanVariance, NormalWeightedMeanVariance, NormalMeanPrecision, and NormalWeightedMeanPrecision. Following the mechanism described above, developers of inference backends could create specific factor nodes for each of these implementations. However, this introduces two problems. First, it exposes an implementation detail (the user should not have to care about the parameterization used for the normal distribution), and it breaks the convenient syntax of Code Block 2, where we could call Normal(x, *σ*). To address these issues, GraphPPL.jl contains an elaborate aliasing system based on keyword arguments that developers can customize to their needs. With this aliasing system, developers can transform Normal(*ν* = 1, *τ* = 1) to NormalWeightedMeanPrecision(1, 1). With this aliasing system, developers can hide implementation details from users and maintain a high-level intuitive interface.

GraphPPL.jl is a backend-agnostic modeling language; therefore, a GraphPPL.jl model does not contain any information concerning the inference process by default. However, some inference backends, like ReactiveMP.jl, require additional information along with the structure of the model to conduct inference. To accommodate these needs, GraphPPL.jl exposes an elaborate plugin system. This system allows inference backend developers to include custom code and information, effectively enabling GraphPPL.jl to gather all necessary information for inference. The plugin required to run inference using the ReactiveMP.jl backend is the variational constraints plugin, which we elaborate on below.

### 4.7. Constraint Specification

In Section 2, we explored the utility of CBFE minimization as an effective method for Bayesian inference. Utilizing the CBFE framework necessitates the user to supply a series of constraints on the variational posterior. These constraints can be seen as additional information embedded within the model’s graph structure. The variational constraints plugin in GraphPPL.jl is specifically designed to handle such scenarios, providing support for the ReactiveMP.jl backend.

GraphPPL.jl features a constraint specification language that integrates seamlessly with the @model macro. This language supports two constraints that can be applied to the variational posterior: Factorization Constraints (FCs) and Functional Form Constraints (FFCs). FCs separate the dependencies among specified variables in the variational posterior [38,39]. For instance, consider a generative model with variables x, y, and z, where we aim to impose a mean-field assumption on these variables in the variational posterior q(x,y,z). The FC q(x, y, z) = q(x)q(y)q(z) would represent this assumption and inject this information into the graph, which can later be utilized by an inference backend. FFCs, on the other hand, restrict the marginal distribution of a specified variable in the variational posterior to a particular functional form. For example, if we want the marginal distribution for x to follow a Beta distribution, we can include the constraint q(x) :: Beta in our constraint specification. This syntax is implemented in the @constraints macro, offering an advanced language for specifying constraints on the generated factor graph for a model.

The constraint language implemented in GraphPPL.jl has one additional feature that harmonizes with the rest of the PPL: By opening **for** code blocks, one can access the constraints for variables in submodels in the factor graph. For example, a mean-field constraint on the *ω*, *κ*, and u variables in every gcv instance in the hgf model of Code Block 5 can be imposed using the constraint specification seen in Code Block 10.


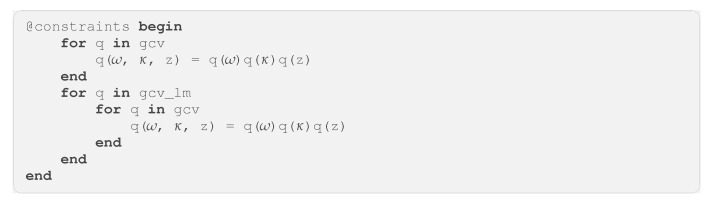

**Code Block 10.** Example constraint specification for an HGF model.

With this syntax, we supply a concise constraint language that still offers significant flexibility and control over the family of variational posterior distributions to consider.

## 5. Inference Example with the ReactiveMP.jl Backend

**Figure 8:**
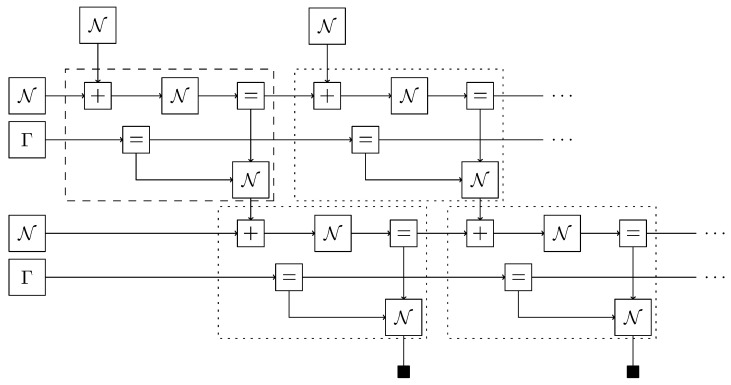
Model used in inference. The dashed box denotes a subgraph that is reused over the entire graph.

In this example, we create a model using GraphPPL.jl with the variational constraints plugin enabled and then perform inference using the ReactiveMP.jl backend. This combination of frontend and backend is accessible in the RxInfer.jl package. We are working with a simple hierarchical state-space model. The FFG of this model is shown in Figure 8. In the FFG diagram, we have omitted constant hyperparameters for distributions to avoid visual clutter. This model describes a random walk with drift, where the random walk also determines the drift. Inside the dotted box is a repeated pattern: two Gaussian distributions are summed, followed by applying another Gaussian distribution, resulting in an observation with a Gaussian likelihood and a Gamma-distributed precision parameter. This model can be used as a nested model in GraphPPL.jl. As seen in Code Block 11, we use the ssm submodel twice, which invokes the ssm_step submodel on every data point. In this way, we can create a complex factor graph in under 20 lines.

Our model assumes a joint dependency between the hidden state drift and the noise in this hidden state drift. However, in our variational posterior distribution, we would like to see these two variables as independent of each other. That is why we impose the factorization constraint q(x_next, y, precision) = q(x_next, y)q(precision) in every copy of the ssm submodel. Code Block 12 shows how to apply this constraint to all copies of the ssm submodel.


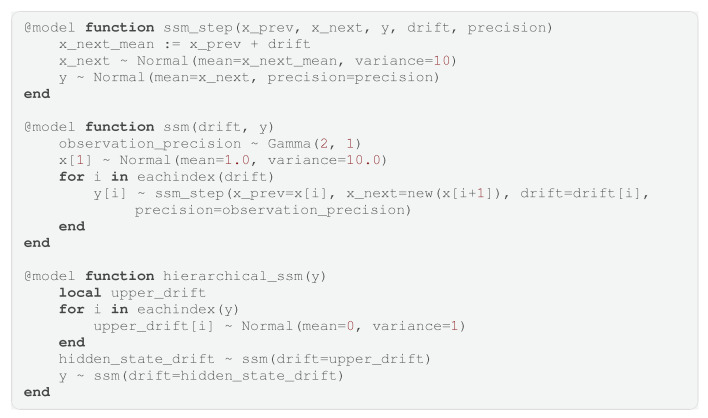

**Code Block 11.** Simple hierarchical state-space model in GraphPPL.jl.

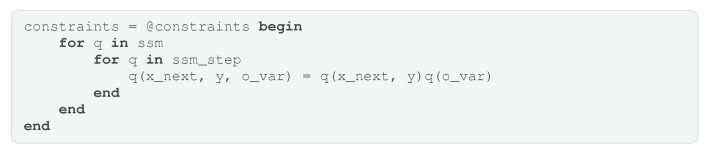

**Code Block 12.** Inference constraints for the hierarchical state-space model.

**Figure 9:**
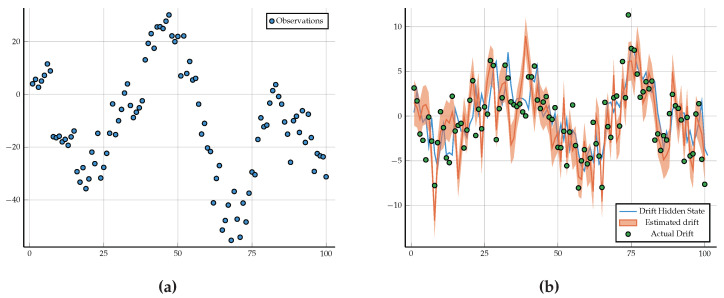
Generated observations and inference result. (**a**) Generated observations. (**b**) Hidden state drift and recovered estimated drift.

To run inference on this model, we first have to generate data. We generated 100 data points, as shown in Figure 9a. We then ran inference using RxInfer.jl by calling the infer function, as seen in Code Block 13. After running inference with RxInfer.jl, we recovered the hidden state hidden_state_drift that drives the change between the hidden states that generate the observations. In Figure 9b, we can see the inference result, showing that we can use GraphPPL.jl with the ReactiveMP.jl backend to automate a sophisticated Bayesian inference process. The code used to generate these images can be found at Github (https://github.com/wouterwln/GraphPPL-demo) (accessed on 18 September 2024). Additional examples can be found in the code blocks in Appendix A or in the RxInfer Examples.


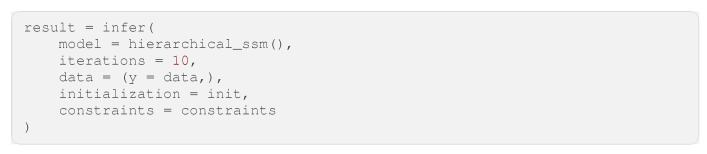

**Code Block 13.** Running inference in the hierarchical_ssm model with generated data.

## 6. Discussion

In this paper, our objective was to design and implement a PPL with the following desiderata/design principles:

- It should be possible to use any GraphPPL.jl model as a submodel in any subsequent model.
- A materialized GraphPPL.jl model should contain all information necessary to perform Bayesian inference. The model should be extendable by backend developers to include additional information.
- A GraphPPL.jl model should look, as much as possible, like the mathematical representation of the generative model, exposing as few implementation details as possible.

In Section 4.4, we introduced modularity, showing how we can use any existing GraphPPL.jl model as a submodel in larger models. This modularity in graphical model construction is versatile and allows for a rich class of models. As seen in Section 5, we can create complex graphical models with readable and interpretable code. The Bayesian Brain Hypothesis [40] suggests that intelligent biological agents are Bayesian reasoning machines, continuously reducing uncertainty about their environments by processing sensory observations. This hypothesis is further supported by the Free-Energy Principle [41], which theorizes that intelligent agents possess a generative model of their environment in their brains. Furthermore, it has been suggested that the main prerequisite for intelligent behavior in biological agents is the hierarchical composition of the agent’s generative model into smaller submodels, each aiming to minimize Bayesian surprise independently [42,43]. This hierarchical structure allows for modularity and scalability in cognitive processes, enabling more efficient and flexible adaptation to new information and environments. With the introduction of GraphPPL.jl, we have introduced a language where the nesting of probabilistic models is at the core of its design philosophy, allowing the design of agents inspired by the Free-Energy Principle.

With the variational constraints plugin, GraphPPL.jl can be enabled to fully specify a CBFE. We have seen that many popular inference algorithms can be written as a minimization of a CBFE [14]. In GraphPPL.jl, we realize this by supplying a generative model with a set of inference constraints (Factorization Constraints and Functional Form Constraints) that together fully define a CBFE to be minimized. However, while Factorization Constraints and Functional Form Constraints allow for an expressive constraint language, these are not the only constraints one could apply to the variational posterior. Examples of constraints that are not covered by GraphPPL.jl are Chance Constraints [44] or joint Functional Form Constraints (Functional Form Constraints that constrain the functional form of joint posterior distributions over certain variables instead of constraining the functional form of a single random variable). While an advantage can be gained by supporting a wider class of constraints, we are unaware of any inference backend that supports these constraints, so no effort has been made to expose these constraints in GraphPPL.jl.

One might be interested in performance issues and analysis with GraphPPL.jl, as well as the availability of model statistics such as model comparison with Bayes factors. We point out that GraphPPL.jl is a language for specifying a model with inference constraints but does not execute the inference procedure. In the existing integration with the RxInfer.jl inference engine, we can analyze the performance of the inference procedure, and since RxInfer.jl approximates the variational free energy, we also have an approximation of the model evidence, which could be used for model comparison.

Future improvements to this work might extend the functionality of the GraphPPL.jl engine, such as having an extended class of constraints available or having an extensive visualization tool with which users can inspect their graphical models.

## 7. Conclusions

In this paper, we have introduced GraphPPL.jl, a probabilistic programming language for graphical models. GraphPPL.jl represents a probabilistic model as a factor graph and uses a custom syntax to efficiently generate factor graphs from high-level user code. With the plugin system, GraphPPL.jl becomes highly extensible. We have shown the extensibility of GraphPPL.jl by implementing the variational constraints plugin, which allows users to define a Constrained Bethe Free Energy instead of only defining a generative model. With this plugin, we have integrated GraphPPL.jl as the frontend of the ReactiveMP.jl inference backend, which minimizes the Constrained Bethe Free Energy using message-passing. This shows the versatility of GraphPPL.jl and the importance of a backend-agnostic, extensible probabilistic programming language.

GraphPPL.jl introduces a mechanism for model nesting, allowing for modular graphical models and, therefore, greatly reducing the cognitive complexity a user is burdened with when creating large graphical models. In short, GraphPPL.jl is an intuitive, powerful, expressive probabilistic programming language that ensures separation between model definition and inference while still providing extensibility and customizability to developers.

## Data Availability

The original contributions presented in the study are included in the article, further inquiries can be directed to the corresponding author.

## Appendix A. Additional Examples

In this appendix, we demonstrate some interesting additional examples of the GraphPPL.jl modeling language.

### Appendix A.1. Recursive Generative Model

In this example, we demonstrate a Quadtree-like structure. A Quadtree is a data structure that recursively divides a two-dimensional space into quadrants. Here, we write a model in GraphPPL.jl inspired by a Quadtree to demonstrate the recursive capabilities of GraphPPL.jl. In our model, we recursively use an aggregate function that combines the intermediate variables of the four quadrants, where the base case is the leaves.

Here, the depth of the model is determined by the size of the input; an 8×8 input results in a model of depth 4, whereas a 128×128 input results in a model of depth 8. At every layer, we have the intermediate representation (i1, i2, i3, and i4), together with all variables that reside in this model, which contains a coarse-grained representation of the output. In this sense, a model like this can be seen as inspired by a Scale-Space [45] method, where every layer represents a coarser, higher-level version of the input.

**Code Block A1.** Recursively defined generative model. Depth is determined by the input size.

### Appendix A.2. Variational Autoencoder

A significant advantage of GraphPPL.jl is that submodels do not have a fixed generative direction in which to be used. This makes the modularity of GraphPPL.jl significantly different from that of Turing.jl. In this example, we demonstrate that by using the neural network architecture defined in Section 4.5, we can easily create a variational autoencoder [46] by making two copies of our encoder–decoder submodel.

**Code Block A2.** Neural network used as both encoder and decoder in the variational autoencoder example.

Materializing this model twice, once in the forward generative direction and once mirrored, gives us a variational autoencoder. Using the modularity of GraphPPL.jl, we can guarantee that the encoder and decoder of this network have the same architecture, and if we change something in this model, we know the that changes will be reflected in both places.

**Code Block A3.** A Variational autoencoder, with the decoder implemented as a mirrored version of the encoder.
